# Potassium Channels Rule over Insulin Release with an Ion Fist

**DOI:** 10.1371/journal.pbio.0040053

**Published:** 2006-01-17

**Authors:** Françoise Chanut

Diabetes is characterized by abnormally high sugar concentration in the blood and urine. The condition can eventually cause kidney and heart failure, blindness, and poor circulation, often requiring amputation of an afflicted limb. In the late 1800s, scientists studying digestion removed the pancreas of a dog and noticed that the dog's urine was laced with sugar. What they and others eventually established is that the pancreas secretes insulin, a hormone that helps tissues pump sugar out of the blood to use as fuel or store for later use. The discovery led to an essentially overnight recovery for millions of diabetics.

Insulin is secreted by β-cells, which are nestled inside small ball-shaped pockets of the pancreas known as the islets of Langerhans. β-cells respond to rising levels of glucose in the blood by releasing insulin, which prevents hyperglycemia. Conversely, when glucose levels fall below a particular threshold, β-cells stop secreting insulin, which prevents the equally dangerous hypoglycemia. In a healthy individual, blood glucose levels stabilize around one gram per liter.

The mechanisms that couple insulin production to blood sugar levels in normal individuals are not fully understood. In a new study, Jonathan Rocheleau, David Piston, and their colleagues show that the free circulation of electric charges among adjacent β-cells, a phenomenon known as electrical coupling, allows the cells to coordinate their response to changing glucose concentrations. [Fig pbio-0040053-g001]

**Figure pbio-0040053-g001:**
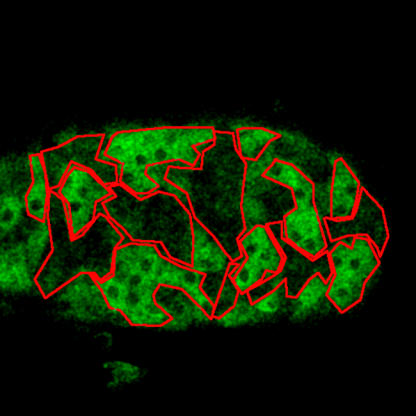
GFP fluorescence within an intact islet marks β-cells that do not have active potassium channels. Regions are outlined that contain cells with either normal or inactive channel activity

All cells in the body maintain an uneven distribution of electric charges—mostly carried by ions such as potassium, sodium, and chloride—across their outer membranes, which are therefore polarized. Changes in membrane polarization act as signals for various cell functions. In β-cells, a reduction of the transmembrane charge difference, called depolarization, triggers insulin release. The molecule that links membrane polarization to insulin release is the ATP-dependent potassium channel. The channel sits at the β-cell's outer membrane and keeps the membrane polarized by maintaining a sharp gradient of potassium distribution across the membrane. As long as the membrane is polarized, β-cells keep insulin trapped inside secretory vesicles. But as β-cells take up glucose, they transform the sugar into ATP, a small energy-carrying molecule that closes the potassium channel. The resulting membrane depolarization causes a massive influx of calcium inside the cells, which in turn allows the vesicles to release insulin to the outside.

When analyzed in culture dishes, isolated β-cells display a wide range of sensitivity to glucose, whereas in the pancreas they release insulin coordinately above a specific glucose threshold. β-cells are tethered together in an islet by gap junctions, areas of their outer membrane that are riddled with intercellular pores through which small molecules circulate freely. This arrangement led to the proposal that ions crossing the gap junctions could harmonize the distribution of electric charges among adjacent β-cells, thereby coordinating their membrane polarization and insulin secretion.

To test this proposal, Rocheleau et al. used transgenic mice whose islets contained a mixture of β-cells with normal potassium channels and β-cells with channels that can't transfer potassium ions. When dispersed in culture, cells carrying the deficient potassium channels were permanently depolarized, and secreted insulin regardless of glucose concentration. But within the islets, they behaved exactly like their normal counterparts: in low glucose concentrations they were polarized, and when glucose concentration reached one gram per liter they became depolarized and took up calcium to the same extent as their normal neighbors. In the presence of a chemical that disrupts gap junctions, however, cells with normal and mutated channels regained their independent responses to glucose.

The authors conclude that the cells carrying an active potassium channel impose their polarized state on neighboring cells, presumably via the free circulation of ions through gap junctions. In a normal pancreas, they propose that a few glucose-resistant cells could clamp others in a polarized state, thereby stamping out natural variations in glucose sensitivity and reducing noise in insulin release.

